# A response to ‘inclusion of the homeless in health equity curricula: a needs assessment study’

**DOI:** 10.1080/10872981.2020.1812262

**Published:** 2020-08-24

**Authors:** Haqeeqat S Gurm, Arbind S Hehar, Arjun S Joshi, Neha K Karthikeyan

**Affiliations:** Faculty of Life Sciences and Medicine, King’s College London, London, UK

**Keywords:** Medical education, homelessness, representation, social care, public health

Dear Editor,

We read with great intrigue this paper by Feldman et al. [1]. It highlighted an important, yet often neglected topic in healthcare education.

As British medical students, it is intriguing to see the parallels in the lack of teaching surrounding homeless populations in healthcare education in America, however it was striking to see that over 20% of students in this study had personal experiences with homelessness. It was a particularly shocking statistic due to our own sheltered experiences in this field, as well as our incredulity that this may be representative of our own cohort. This could be indicative of the very nature of the issue; it is one that stems from ignorance. The failure of medical education to shed light on this ignorance is detrimental to say the least, not only to the future of clinical practice but also in protecting one of the most vulnerable populations. Questions need to be asked how health systems founded to strive for equal healthcare for all irrespective of class, creed or colour have failed to mobilise adequate support for the homeless. The answers to these questions can only be found through adequate representation and further research into the needs and wants of this population.

The importance of receiving specific teaching on the homeless population cannot be understated. There is evidence showing links between social determinants of health and patient outcomes [2]. It is established that factors such as employment, socioeconomic circumstances and urbanisation all have a role to play in health yet, there are key gaps in the curricula when learning how these fit into managing patients in the real world. We are not explicitly taught how to create a discharge plan for a homeless patient, how to maintain their engagement with services and where to direct them for further support. Further to this, in order to provide holistic care, one must learn to navigate social issues the patient may present with e.g. getting them help with shelter, mental health services and other necessities. This is especially pertinent in the current pandemic where organisations that normally help with these may be working under reduced capacity yet there is very little emphasis from academic institutions on the importance of supporting this patient group with these unique struggles.

In striving to understand these struggles, exposure to caring for homeless populations could potentially help foster empathy for their situations and change the negative attitudes, stereotypes and stigmas often harboured towards the homeless, as shown possible in a study by Walsh et al. [3] This is also corroborated in the article as those with prior exposure to homelessness had more positive attitudes in caring for this population (higher HPATHI scores). Though didactic teaching on the topic can be helpful, it can also serve to homogenise a patient population defined by individual tribulations, each with a story to tell that guides the personalised care they need. One way this can be addressed is by encouraging student involvement in programmes such as ‘Street Medicine’, a well-known concept in the US but one that requires expansion in the UK. As prefaced by ‘FG B, Student E’ [1], this would be an effective teaching method as healthcare students would be learning from the experienced leaders of the roaming medical teams and just as with other clinical placements, learning is often maximised when thrown into the deep end and dealing directly with patients. An important component of the delivery of this teaching not touched on in the article is having debrief sessions after the placements. This has shown to consolidate learning in these environments as well as, with the aid of supervisors, challenging unconscious biases that may have been exposed during their experiences [4].

Homeless people often do not seek out necessary healthcare, therefore it is the responsibility of healthcare professionals to seek them out. To do so requires, first and foremost, teaching within medical training to better prepare the future workforce. This appetite for a greater emphasis on homeless populations in healthcare curriculums from American healthcare students is mirrored by their British counterparts [3], including the authors themselves.
